# Manifestation of “Wing‐Beating” Tremors Along With Appearance of the “Giant Panda” Sign on Brain MRI in Neuro Wilson's Disease Due to Treatment Failure: A Case Report

**DOI:** 10.1002/ccr3.70456

**Published:** 2025-04-21

**Authors:** Rupal Vikas Dosi, Kush Pankaj Shah, Jeel Narendra Sarvaiya, Dakshi Jignesh Kadakia, Dax Rajendra Patel, Aarushi Mishra

**Affiliations:** ^1^ Baroda Medical College and SSG Hospital Vadodara India; ^2^ B.J. Medical College Ahmedabad India; ^3^ Danylo Halytsky Lviv National Medical University Lviv Ukraine

**Keywords:** d‐penicillinamine, giant panda sign, Kayser–Fleischer ring, Wilson's disease, wing‐beating tremor, zinc acetate

## Abstract

We report a patient of neuro Wilson's disease who presented with “wing‐beating” tremors and “giant panda” sign on magnetic resonance imaging, 1 year after initiation of treatment with zinc acetate. We focus on the importance of initiating copper chelator therapy as the first line treatment as it improves the prognosis and prevents irreversible complications.

**Abbreviations**
MRI: magnetic resonance imaging
NWD: neuro Wilson's disease

## 1 Introduction

Wilson's disease is a metabolic dysfunction disorder with deranged copper metabolism and is inherited in an autosomal recessive pattern. ATP7B is the primarily affected gene, which alters biliary copper excretion, leading to copper buildup in the liver parenchyma and basal ganglia of the brain [1]. Rarely, involvement of both the liver and brain has been reported [2]. Neuro Wilson's disease (NWD) occurs when copper deposition primarily deposits in the basal ganglia and other parts of the brain and manifests as dysarthria, dystonia, tremors, and incoordination. The epidemiology of NWD is not well documented, although it is more commonly reported in regions with higher rates of consanguineous marriages.

Our patient presented with worsening tremors, a new onset of “wing‐beating” tremors, and a “giant panda” sign on brain MRI 1 year after the initiation of zinc acetate. The patient had a high existing copper store in the body, which led to worsening of the patient's condition. The presence of “wing‐beating” tremors together with a “giant panda” sign alongside a high Leipzig score, due to treatment failure is a very rare finding and makes our case unique and interesting. Only a few cases have been reported in the literature that show the presence of both findings in a single patient. No similar literature has reported the presence of both findings after the initiation of treatment.

We report this case with the aim of highlighting the rare features of the condition that develop due to the advancement of the condition by treatment failure. We focus on the importance of early initiation of appropriate treatment protocols to prevent disease progression.

## 2 Case History and Examination

A 26‐year‐old Hindu Indian female patient working at a call center initially presented to the outpatient department of our hospital 12 months ago with the complaints of low‐grade bilateral upper limb and lower limb tremors for 4 months. The patient presented to the hospital when the tremors hindered her daily activities. It aggravated on movement and relieved at rest. No significant previous medical history was noted. No similar complaints were present in any other family member. Her menstrual and obstetric history revealed delayed menarche at the age of 16 years with the usual menstruation cycle of 3–6 months and G_0_P_0_A_0_L_0_. No history of the use of copper utensils at home was given. Ocular examination findings revealed the presence of Kayser–Fleischer rings in the peripheral cornea in a ring pattern. Serum glutamic oxaloacetic transaminase, glutamate pyruvate transaminase, alkaline phosphatase, and ceruloplasmin were investigated. Serum ceruloplasmin was raised while others were within reference range. Twenty‐four hour urinary copper excretion also turned out to be raised. No other differential diagnoses were considered. The patient was diagnosed with NWD and was prescribed zinc acetate 50 mg twice a day to reduce copper absorption, trihexphenidyl 2 mg twice a day, and etizolam 0.5 mg with propranolol 20 mg fixed‐dose combination once a day to control tremors.

The patient now presented with asymmetrical bilateral upper limb tremors, low‐grade left lower limb tremors, neck and face tremors, and dystonia of the neck and face on turning the face towards the left side for 12 months. She complained of worsening of the symptoms along with new onset dysarthria for 1 month.

**FIGURE 1:**
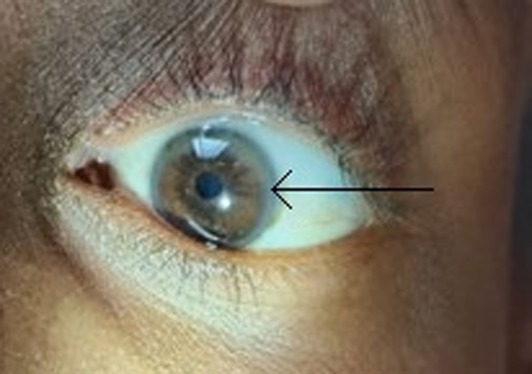
Kayser–Fleischer ring in descement layer of cornea.

On examination, the patient was well oriented to time, place, and person. Her vital signs were normal. Physical examination: (1) On ocular examination, Kayser–Fleischer rings were noted (Figure 1). (2) Neurological examination revealed classical “wing‐beating” tremors bilaterally in the upper limbs (left > right) and the left lower limb. The tremors were present at rest with aggravation on movement, especially on arm abduction with elbow flexion. They were low in frequency and high in amplitude. A generalized hypertonia with cogwheel phenomena in the muscles of both upper limbs (left > right) and left lower limb were noted with gross incoordination in upper limbs. They were associated with “NO‐NO” neck tremors and face and neck dystonia on turning the face on the left side. (3) Abdominal examination was unremarkable. Liver and spleen were nonpalpable.

## 3 Investigations and Treatment

The laboratory investigations are described in Table 1.

**TABLE 1:** Laboratory investigations.

Lab parameters	Values	Reference range
Hemoglobin	12.70 g%	12–16 g%
Serum glutamic oxaloacetic transaminase	40 U/L	10–40 U/L
Serum glutamate pyruvate transaminase	28 U/L	12–38 U/L
Serum alkaline phosphatase	339 U/L	25–100 U/L
Serum ceruloplasmin	6.3 mg/dL	16–35 mg/dL
24‐h urinary copper excretion	223.5 μg/24 h	15–60 μg/24 h

Abbreviations: dL, deciliter; g%, gram percentage; L, liter; mg, milligram; U, units; μg, microgram.

**FIGURE 2:**
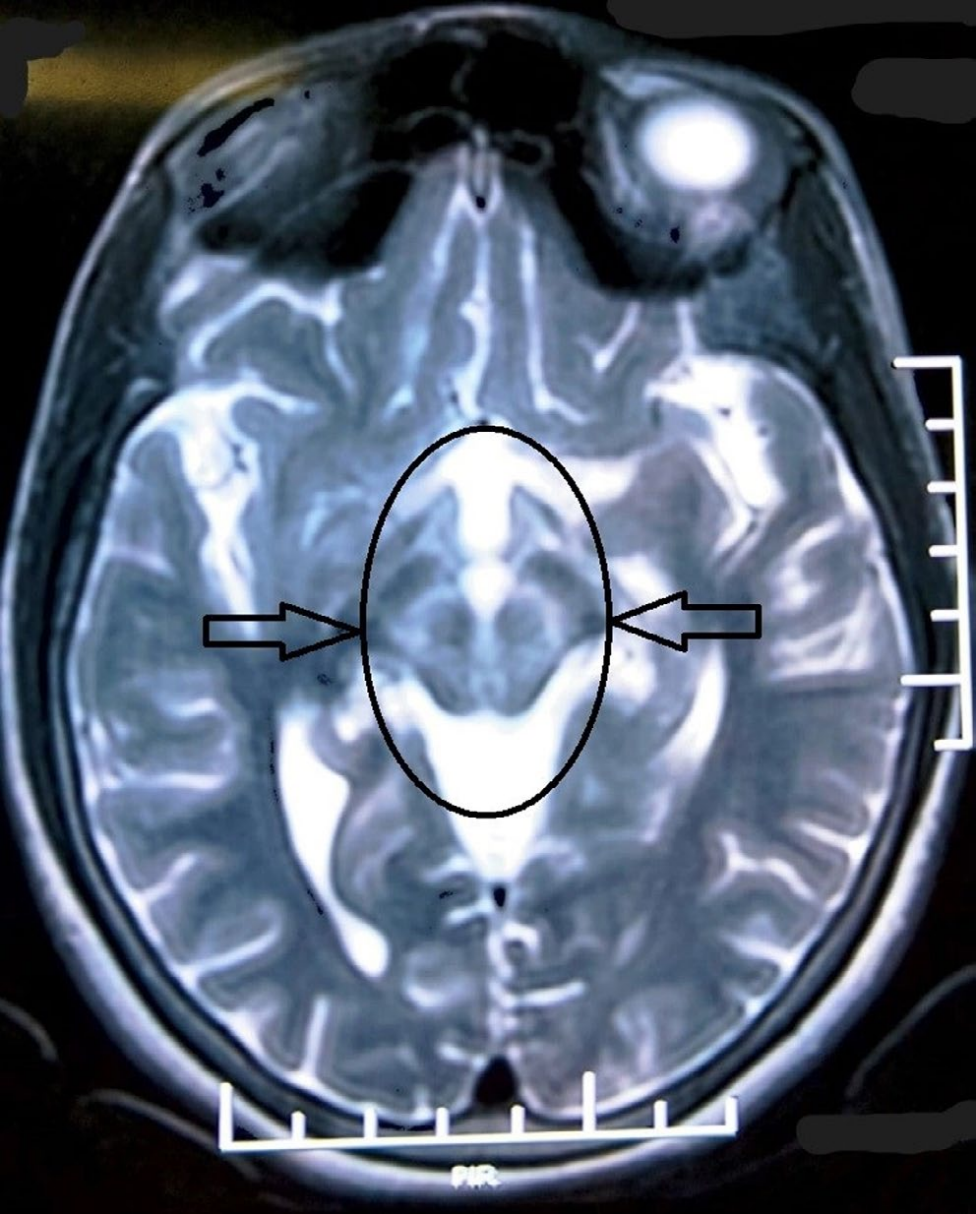
“Giant panda” sign is noted in the midbrain on MRI.

Ultrasonography of the abdomen revealed a normal sized liver with normal echogenicity, nondistended spleen, and absent free fluid in the abdomen. T2‐weighted brain magnetic resonance imaging (MRI) revealed a “giant panda” sign (Figure 2) with bilaterally symmetrical high signal intensities at B/L tegmentum except at the red nuclei. These MRI findings were absent 1 year ago when the patient was initially diagnosed with NWD. A Leipzig score of 8 was calculated, and appropriate medications were initiated. Worsening tremors in a known case of NWD who is compliant with medication is most likely due to treatment failure.

Hence, the patient was diagnosed with neurological exacerbation in a known case of NWD due to treatment failure.

The patient was initiated on pharmacological intervention with oral d‐penicillamine 250 mg four times a day, along with oral pyridoxine 10 mg thrice a day and oral zinc acetate 50 mg twice daily, along with oral tetrabenazine 25 mg thrice daily and oral propranolol 40 mg once daily. The patient was kept in the medical ward for 7 days.

## 4 Outcomes and Follow‐Up

The patient, during her indoor admission, experienced significant improvement in tremors and dysarthria. After discharge, the patient was advised regular follow‐up. The patient was adherent to drugs, and significant improvement in tremors and dysarthria was noted with every 2 monthly follow‐up visits. Absent “wing‐beating” tremors were observed eventually. No significant adverse drug reactions were developed.

## 5 Discussion

Wilson's disease is a genetic disorder inherited in an autosomal recessive pattern. Mutation foci lie within the ATP7B gene on chromosome 13, which encodes the copper transporting P‐type ATPase protein and is responsible for copper excretion from the body. When this protein is mutated, copper accumulates and leads to oxidative stress due to free radical formation in the liver, brain, and other organs [1, 3]. Symptoms of NWD usually present in the second or third decade of life with dysarthria, dystonia, tremor, choreiform movements, and incoordination. Zhang et al. also reported nocturnal enuresis along with other symptoms [4]. “Wing‐beating” tremors develop due to copper accumulation in the dentato‐rubro‐thalamic pathway which forms an important connection between the cerebellum and thalamus. Disruption of this pathway leads to incoordination of motor movements. NWD should be suspected and considered as a differential diagnosis, particularly in the presence of neurological symptoms like tremors, dysarthria, anxiety, and depression; liver symptoms like jaundice, abdominal pain and right hypochondrium abdominal swelling; and the presence of Kayser–Fleischer rings in the eyes bilaterally.

On MRI of the brain, the characteristic “giant panda” sign can be noted in only 14.3% of patients [5]. This appears in the midbrain due to hyperintensities developed by gliosis and edema in the bilateral tegmentum except for red nuclei forming the eyes, normal intensity in the lateral portion of the pars reticulata of the substantia nigra forming the ears, and hypointensity in the superior colliculus developed because the paramagnetic properties of copper influence the local magnetic field, forming the mouth [6]. The “Miniature panda” sign and “Trident” sign are other findings noted in the brain MRI by Jacob et al. and Parekh and Agrawal, respectively [6, 7]. The presence of these findings is an important indicator of worsening conditions, suggesting a change in the treatment protocol. These findings are also highly impactful for accurate diagnosis of NWD in patients having these as their primary clinical findings.

A definitive diagnosis of Wilson's disease is made by a standardized score obtained via the Leipzig scale. Parameters scored in the scale include the presence of Kayser–Fleischer rings, neurological symptoms or brain MRI findings, Coombs‐negative anemia, reduced levels of serum ceruloplasmin, increased levels of 24‐h urinary copper excretion, increased total liver copper levels, and presence of genetic mutations. A total score of ≥ 4 is confirmatory for Wilson's disease.

Our patient initially received zinc acetate therapy alone, which reduced the new copper absorption from the gut but failed to excrete existing copper stores, causing treatment failure. Hence, the standard treatment protocol in Wilson's disease is copper chelator therapy, which excretes existing copper stores indicated for the rest of life, most commonly d‐penicillamine owing to its affordability. It is prescribed along with prophylactic pyridoxine (B_6_) to prevent optic neuritis due to pyridoxine deficiency. Worsening of neurological symptoms and a new appearance of myasthenia gravis are occasionally observed due to d‐penicillamine [8, 9]. Alternatively, trientine and tetrathiomolybdate can be prescribed when d‐penicillamine is not tolerated. Zhou et al. provided evidence for faster treatment and less aggravation of symptoms with the use of dimercaptopropane sulfonate and dimercaptosuccinic acid as compared to D‐penicillamine [10]. Zinc acetate is prescribed as an adjuvant for effective management of patients. Treatment of NWD in the early stages leads to reversal of all the signs and symptoms.

## 6 Conclusions

Our patient developed “wing‐beating” tremors and a “giant panda” sign on a brain MRI 12 months after the initiation of zinc acetate as her primary treatment for NWD. She was managed by identifying these findings and beginning copper chelator therapy. With our case, we emphasize the rare clinical and radiological findings of NWD that appear due to treatment failure, noncompliance, and in the later stages of the condition, alongside addressing the importance of initiating an appropriate treatment protocol. Knowledge about these features will also help to speed up the diagnosis in cases where patients present in the later stages of the disease.

## Author Contributions

**Rupal Vikas Dosi:** conceptualization, formal analysis, project administration, resources, supervision, validation, writing – review and editing. **Kush Pankaj Shah:** data curation, methodology, visualization, writing – original draft, writing – review and editing. **Jeel Narendra Sarvaiya:** formal analysis, project administration, writing – original draft, writing – review and editing. **Dakshi Jignesh Kadakia:** data curation, methodology, writing – original draft. **Dax Rajendra Patel:** conceptualization, methodology, visualization, writing – review and editing. **Aarushi Mishra:** conceptualization, formal analysis, validation, writing – review and editing.

## Disclosure

Permission from all authors has been obtained for publication of final version of this manuscript. All authors have agreed to be accountable and responsible for all aspects of this work.

## Ethics Statement

The authors have nothing to report.

## Consent

Written consent was obtained by the patient.

## Conflicts of Interest

The authors declare no conflicts of interest.

## Data Availability

The authors have nothing to report.
